# Microplastic-Assisted Removal of Phosphorus and Ammonium Using Date Palm Waste Derived Biochar

**DOI:** 10.3390/toxics11110881

**Published:** 2023-10-26

**Authors:** Munir Ahmad, Muhammad Imran Rafique, Mutair A. Akanji, Hamed Ahmed Al-Swadi, Muhammad Usama, Mohammed Awad Mousa, Mohammad I. Al-Wabel, Abdullah S. F. Al-Farraj

**Affiliations:** Soil Sciences Department, College of Food & Agricultural Sciences, King Saud University, P.O. Box 2460, Riyadh 11451, Saudi Arabia; mrafique@ksu.edu.sa (M.I.R.); makanji@ksu.edu.sa (M.A.A.); halswadi@ksu.edu.sa (H.A.A.-S.); usama.chaudharyuaf@gmail.com (M.U.); awad.mohammed333@yahoo.com (M.A.M.); malwabel@ksu.edu.sa (M.I.A.-W.); sfarraj@ksu.edu.sa (A.S.F.A.-F.)

**Keywords:** polyethylene, polyamide, microplastics, eutrophication, sorption mechanism, vector

## Abstract

Microplastics (MPs) are emerging environmental pollutants worldwide, posing potential health risks. Moreover, MPs may act as vectors for other contaminants and affect their fate, transport, and deposition in the environment. Therefore, efficient and economical techniques are needed for the removal of contemporary MPs and contaminants from the environment. The present research study investigated the sorption of phosphorus (P) and ammonium (NH_4_^+^) onto date palm waste-derived biochar (BC) from an aqueous solution in the presence of polyamide (PA) and polyethylene (PE) MPs. The BC was prepared at 600 °C, characterized for physio-chemical properties, and applied for P and NH_4_^+^ removal via isotherm and kinetic sorption trials. The results of the sorption trials demonstrated the highest removal of NH_4_^+^ and P was obtained at neutral pH 7. The highest P sorption (93.23 mg g^−1^) by BC was recorded in the presence of PA, while the highest NH_4_^+^ sorption (103.76 mg g^−1^) was found with co-occurring PE in an aqueous solution. Sorption isotherm and kinetics models revealed that P and NH_4_^+^ removal by MP-amended BC followed chemisorption, electrostatic interaction, precipitation, diffusion, and ion exchange mechanisms. Overall, co-existing PA enhanced the removal of P and NH_4_^+^ by 66% and 7.7%, respectively, while co-existing PE increased the removal of P and NH_4_^+^ by 55% and 30%, respectively, through the tested BC. Our findings suggested that converting date palm waste into BC could be used as a competent and economical approach to removing P and NH_4_^+^ from contaminated water. Furthermore, microplastics such as PE and PA could assist in the removal of P and NH_4_^+^ from contaminated water using BC.

## 1. Introduction

The formulation of plastic was considered an innovative step in material science, and as a result, the production and utilization of various kinds of plastic products became part of daily life routines [1]. PlasticsEurope [2] documented the highest plastic production of about 8.3 billion tons worldwide, and about 20% of this produced plastic is recycled, and the remaining 80% is piled up in aquatic (rivers and oceans) and soil environments [3]. As a result of weathering and various environmental activities, including physio-chemical and biological actions, the gathered plastic waste in the environment is transformed into smaller fragments and particles less than 5 mm in diameter, forming microplastics (MPs) [4]. Depending on their source of origin, two types of MPs are present, which include primary MPs (less than 5 mm diameter) and secondary microplastics (originated from the degradation of large plastics or primary MPs) [5]. As a consequence of such environmental activities and processes, MPs possess diverse physio-chemical properties such as smaller particle size, pollutant carrying (heavy metals and antibiotics), stronger hydrophobicity, stable chemistry, and large specific surface area. Also, MPs act as a vector and can attach metal ions and other organic pollutants from their surroundings in an aqueous environment to their surface because of their large surface area-to-volume ratio and smaller size [6,7,8]. Biofilms formed on the surface of microplastics can affect and ultimately enhance their capacity for the sorption of pollutants [9].

Certain natural sources and anthropogenic activities such as mineralization, dumped industrial and domestic waste, landfill leachate, surface runoff from fertilizer and agricultural waste, livestock, and farmyard waste are responsible for the excessive accumulation of nitrogen (N) and phosphorus (P) contents in an aqueous environment [10,11]. Excessive contents of these nutrients in an aqueous environment can result in the accumulation of algae blooms, oxygen-limited conditions, anoxic and acidic environment, resulting in toxin production, deprivation of plant diversity, food web disruption, and health issues [12]. Therefore, it is very important to formulate and employ environmentally friendly, cost-effective, and practical techniques to remove excessive contents of N (NH_4_^+^) and P in water bodies. Among the several utilized techniques, including ion exchange, biological conversion electrodialysis, and chemical precipitation, adsorption has been classified as the most effective, easy to practice, and feasible technique to recover or remove NH_4_^+^ and P from aqueous systems [13]. Adsorbents such as ion exchange resin, zeolites, and activated carbon have the potential to remove nutrients; however, these are expensive raw materials [13]. Therefore, the lowest cost, eco-friendly, easily available adsorbent is a prerequisite to remove nutrients such as N and P.

Biochar (BC), which is a carbon-enriched spongy solid material, is obtained from the controlled burning of organic waste [14]. It has gained the attention of researchers for use as an adsorbent because of its exclusive characteristics such as diverse functional groups, porous surface, huge surface area, high cation exchange capacity, and sorptive and hygroscopic properties [15]. Previous studies reported that BC showed excellent adsorption of NH_4_^+^ and P ions in the aqueous phase [16,17]. In a study, Pratiwi et al. [18] reported that BC produced from rice husk has the potential to adsorb NO_3_^−^ and NH_4_^+^. Likewise, the adsorption of NH_4_^+^ has been reported in various studies by using BC derived from various feedstocks such as *Chamaecyparis obtusa* [19], avocado seed [20], corn cob [21], rice husk, wood [22] and sugarcane bagasse [23]. In a review article, Micháleková-Richveisová et al. [24] documented excellent PO_4_^3^ adsorption capacities by the application of BC derived from different feedstocks such as corn cob, activated rice husk ash [25], rape, and Chinese garbage [26], canola straw, soybean straw, peanut straw [27], eggshell, rice straw [28], and cow dung [29]. In another study, the outcomes revealed that magnesium-doped biochar showed an improvement in the sorption ability for phosphorous [30]. Additionally, scientists have reported the combined application of BC and MPs, particularly polyethylene (PE), has the potential to adsorb and remove NH_4_^+^ from an aqueous environment [31]. Additionally, utilization of BC in mitigating eutrophication and N leaching in water resources has also been documented, which concludes that integrated application of BC along with MPs such as polyethylene (PE) and polyamide (PA) could result in higher sorption of NH_4_^+^ and P in the liquid phase. Therefore, the current study focused on the removal of NH_4_^+^ and P from an aqueous system in the presence of co-existing MPs (PE, PA) through sorption batch trials.

## 2. Materials and Methods

### 2.1. Synthesis and Characterization of Biochar

Date palm fiber waste was collected, washed with tap water, air-dried, and crushed into small pieces for pyrolysis. Afterward, the biomass was placed in stainless steel boxes and kept in a muffle furnace (Wisetherm FH14, Bahnhofstr, Saarbrücken, Germany) at 600 °C at 5 °C per min heating rate for 3 h to produce biochar. The produced BC was collected, cooled at room temperature, ground, and stored after sieving with a 0.53 mm size sieve in an air-free bag and tagged as BC. The BC was characterized for its chemical and surface characteristics by following standard procedures [32,33]. A NaCl solution of concentration 0.10 mol L^−1^ was used to determine the pH at the point of zero charge (pHpzc). Briefly, different NaCl solutions of 0.10 mol L^−1^ concentration with initial pH values of 2 to 12 were prepared, and 0.5 g of BC was suspended in these solutions. The suspension was shaken for 24 h at room temperature, the final pH of the suspension was determined, and pHpzc was estimated. A scanning electron microscope (SEM: EFI S50 Inspect, The Netherlands) was used to determine the morphology of the biochar surface, while the composition of minerals in produced BC by employing an X-ray diffraction technique with a Maxima XRD-7000 (Shimadzu, Kyoto, Japan) was determined. Brunauer–Emmett–Teller (BET) theory was used to analyze the pore volume and surface area of the produced BC. An elemental analyzer (PerkinElmer 2400 CHNS/O series II analyzer, Norwalk, CT, USA) was utilized to interpret the composition of elements in biochar, and the Fourier transform infrared spectroscopy (FTIR) technique (VERTEX-70, Bruker-USA, Billerica, MA, USA) was used to determine functional groups associated with the biochar surface.

### 2.2. Microplastics Preparation

Polyamide (PA) pellets and polyethylene (PE) beads were crushed and ground using a cryogenic grinding process into smaller pieces and stored in a deep freezer [34]. Later on, the suspended plastic particles were collected in a glass beaker and dried. Microplastics, particularly fine particles with a size range of 0–10 μm, were collected.

### 2.3. Sorption Experiments

#### 2.3.1. Effect of Solution pH

The effects of initial pH on the sorption of nitrogen (NH_4_^+^) and phosphorus (P) by the synthesized BC and MPs were observed at adjusted initial solution pH values of 5, 7, and 9. A 100 mg L^−1^ solution of NH_4_^+^ and P was prepared separately using analytical grade NH_4_Cl and KH_2_PO_4_ source compounds. Adsorbents (BC and MPs) were added in 25 mL of 100 mg L^−1^ NH_4_^+^ and P solutions at 1 g L^−1^ separately. Samples were placed in a reciprocal shaker and shaken for 24 h at a constant speed of 140 rpm, followed by filtration with filter paper (Whatman 42). Filtrate was collected, and the remaining NH_4_^+^ and P concentrations were analyzed by UV-visible light spectrophotometer (Lambda EZ 150) following SEPA [35] and Soltanpour and Workman [36] methods, respectively.

The sorption of NH_4_^+^ and P was calculated using Equation (1) (Foo and Hameed, 2010).

(1) $$qe=\frac{\left(Co-Ce\right)\times V}{m}$$

Here, Co represents the initial concentration of NH_4_^+^ and P solutions (mg L^−1^), the concentration at the equilibrium of NH_4_^+^ and P is indicated by *Ce* (mg L^−1^), V is the solution volume, m indicates adsorbent mass and qe represents the adsorbed concentration of NH_4_^+^ and P at equilibrium.

#### 2.3.2. Kinetics Batch Experiments

Kinetics studies were conducted to observe the reaction rate for the NH_4_^+^ and P sorption by BC and MPs. A 100 mg L^−1^ solution concentration and optimum pH value (7) were selected based on previous adsorption batch studies with an adsorbent dose of 1 g L^−1^. Samples were placed in a reciprocal shaker at a constant shaking speed of 140 rpm and withdrawn after regular time intervals of 0, 30, 60, 120, 240, 480, 960, and 1440 min. After the filtration of samples, the filtrate was analyzed for P and NH_4_^+^ concentration using a UV-visible light spectrophotometer (Lambda EZ 150). The sorption capacity of the adsorbents was calculated using Equation (1). Further, various kinetic models such as power function, first-order (Equation (2)), second-order (Equation (3)), pseudo-first-order (Equation (4)), pseudo-second-order (Equation (5)), Elovich (Equation (6)), and intraparticle diffusion (Equation (7)) were used to interpret and analyze these experimental data.

(2) $$I_{n}q_{t}=I_{n}q_{o}-k_{1}t$$

(3) $$\frac{1}{q_{t}}=\frac{1}{q_{0}}-k_{2}t,$$

(4) $$I_{n}=\left(q_{e}-q_{t}\right)=I_{n}q_{e}-{k^{\prime }}_{1}t$$

(5) $$\frac{t}{q_{t}}=\frac{1}{{k\prime }_{2}{q^{2}}_{e}}+\frac{1}{q_{e}}t$$

(6) $$q_{t}=\frac{1}{\beta }\;I_{n\left(\alpha \beta \right)}+\frac{1}{\beta }\;I_{nt},$$

(7) $$q_{t}=c+k_{id}t^{0.5}$$

The adsorbed concentrations of P and NH_4_^+^ at time t and 0 min are indicated with $q_{t}$ and $q_{o}$, respectively (mg g^−1^), time interval is indicated with *t*, $k_{2}$ and $k_{1}$ are the second and the first-order constant rate, respectively, $q_{e\;}$ means the sorption of equilibrium (mg g^−1^), ${k^{\prime }}_{2}$ and ${k^{\prime }}_{1}$ are the rate constants of pseudo-second order and pseudo-first-order, respectively; the initial sorption rate is indicated as α (mg g^−1^min), the sorption constant is indicated as β, the rate constant is indicated with *b*, the coefficient rate value is indicated as $k_{f}$ (mg g^−1^ min^−1^), apparent diffusion rate constant is indicated with $k_{id}$ ([mg g^−1^]^−0.5^) c indicated the diffusion constant.

#### 2.3.3. Equilibrium Batch Studies Isotherm

To interpret the BC and MPs application effects on NH_4_^+^ and P sorption in the aqueous phase, sorption experiments were performed in relation to initial concentration at a constant pH value and contact time. The adsorption isotherm of NH_4_^+^ and P sorption by BC and MPs was observed at variable initial concentrations of adsorbent (0, 5, 10, 25, 50, and 100 mg L^−1^). The amount of NH_4_^+^ and P sorbed onto BC and MPs was calculated using Equation (1). Interpretation of experimental data was determined using the application of various adsorption isotherm models such as Dubinin–Radushkevich (Equation (8)) adsorption isotherm models (Dubinin and Radushkevich, 1947), Temkin (Equation (9)) (Temkin, 1940), Freundlich (Equation (10)) (Freundlich, 1906) and Langmuir (Equation (11)) (Langmuir, 1916) described below:

(8) $$q_{e}=q_{D}exp\left(-B_{D}[{RTI}_{n\;}(1+\frac{1}{C_{e}}{)]}^{2}\right)$$

(9) $$q=\frac{RT}{b}In(AC_{e})$$

(10) $$q_{e}=K_{F}{C_{e}}^{1/n}$$

(11) $$q_{e}=\frac{Q_{L}C_{e}K_{L}}{1+K_{L}C_{e}}$$

where $K_{L}$ is the equilibrium constant of Langmuir sorption (L mg^−1^), maximum adsorption capacity is indicated as $Q_{L}$ (mg g^−1^), Freundlich sorption capacity constant is indicated as $K_{F}$ (L g^−1^), Freundlich intensity constant is *n*; the universal gas constant is R (8.314 J K^−1^mol^−1^), absolute temperature is indicated as T, A (L mg^−1^) indicates the constant of binding, b indicates the heat during adsorption, $q_{D}$ is the maximum adsorption capacity (mg g^−1^), and the mean free energy of sorption is indicated by $B_{D}$.

## 3. Results and Discussion

### 3.1. Characterization of BC

The results of the chemical, ultimate, and surface properties of BC are presented in Table 1. The produced BC yield was 31.04%. The pH of biomass (BM) was 7.87, while that of BC was 10.66, indicating the removal and addition of acidic and basic functional groups, respectively [37]. Likewise, the EC of the BC was higher (1.51 dS m**^−^**^1^) compared with the BM (0.80 dS m**^−^**^1^), suggesting the condensation of the basic functional groups and cationic species. The pH_pzc_ was found to be 7.25 for BM and 11.01 for BC. Compared with BM (6.83%), lower moisture content was found in BC (2.52%). Ash and fixed carbon contents were significantly increased, while volatiles were decreased with the pyrolysis process. The increment in fixed carbon and ash contents could be due to the accumulation of mineral compounds and an improved degree of carbonization [38]. The produced BC showed a substantially higher surface area (260.05 m^2^ g**^−^**^1^) than BM (1.62 m^2^ g**^−^**^1^). Likewise, the pore volume was increased with the pyrolysis process, and the pore size was reduced. The results of elemental composition analysis showed an increment in N and C and a reduction in the contents of H and O with the pyrolysis process. The C content was 63.72% in BC, which agreed with the proximate analysis results. In contrast to BM (0.79 and 1.45, respectively), the ratios (molar) of O/C and H/C were lower in BC (0.41 and 0.10, respectively). The reduction in both O/C and H/C molar ratio as a consequence of the pyrolysis process was due to improved aromaticity degree and maturation [39].

**Figure 1:**
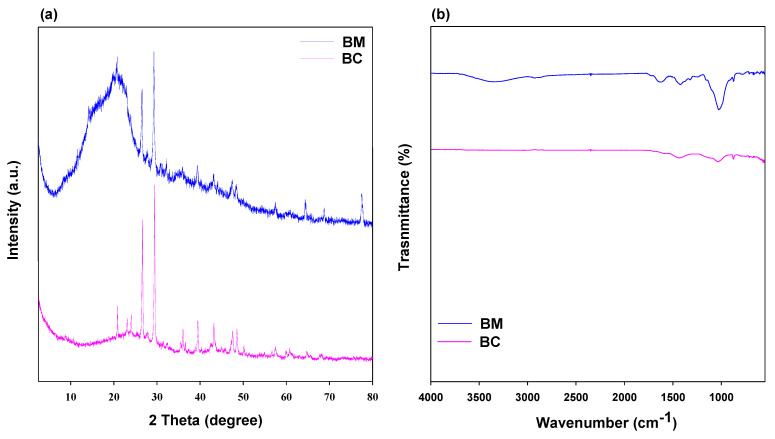
X-ray diffraction analysis (**a**) and Fourier-transform infrared spectroscopy (**b**) analysis of date palm waste (BM) and its derived biochar (BC).

**Figure 2:**
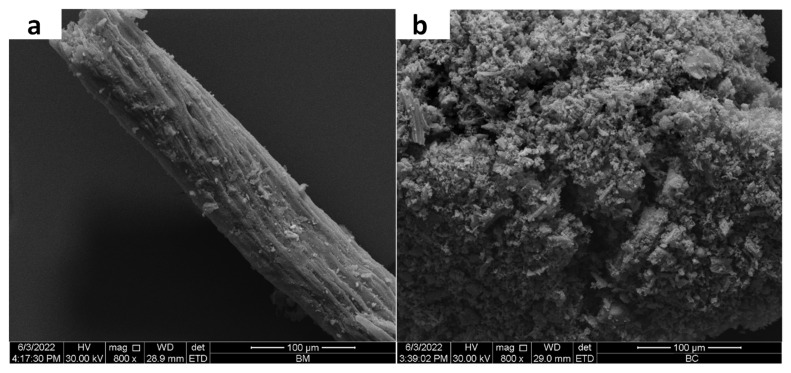
Scanning electron microscopy images of date palm waste (**a**) and its derived biochar (**b**).

The XRD and FTIR analyses of the charred material and raw BM are shown in Figure 1. XRD patterns of BC and BM showed the occurrence of different inorganic and crystalline minerals (Figure 1a). The presence of peaks at 20.88° and 26.68° showed an abundance of SiO_2_ in both materials. A sharp peak around 29° indicated the presence of CaCO_3_ in the materials; however, it was sharper in BM compared with BC. Peaks at 26.4° could be due to the presence of KCl in BM and BC. Additionally, a moderate peak MgO was found in BC. The FTIR spectra in Figure 1b showed that the broad band (3300–3400 cm**^−^**^1^) indicated O–H bonding associated with water molecules in the BM, while it disappeared during pyrolysis and was absent in BC [40]. The bands around 1550–1600 cm**^−^**^1^ showed the presence of hydroxyl groups [41], while the bands near 1400 cm**^−^**^1^ might be owed to the stretching of C–H bonds. In BM, the clear bands at 1000 cm**^−^**^1^ ascribed the stretching of C–O–C as a consequence of polysaccharide cellulose material present in it; however, this band was reduced in BC due to pyrolysis. The surface morphologies of BM and BC, as examined by SEM, are shown in Figure 2. It was observed that the surface of BM was smooth and crystalline (Figure 2a), whereas the surface of BC was rough, porous, and amorphous (Figure 2b).

### 3.2. Sorption Experiments

#### 3.2.1. Effects of pH on Sorption of P and NH_4_^+^

**Figure 3:**
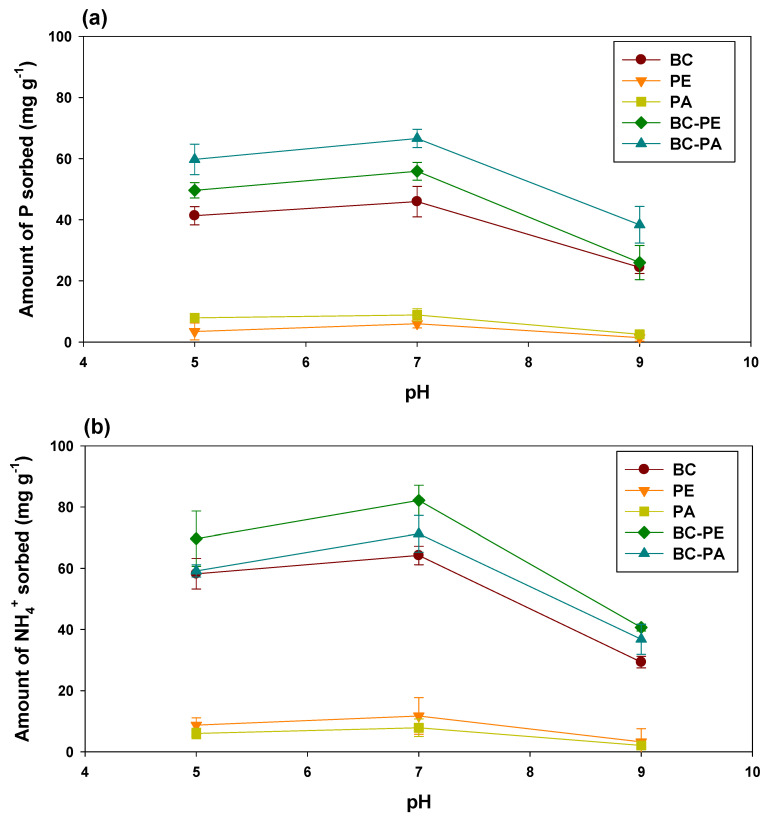
Effects of initial solution pH on the sorption of (**a**) phosphorus (P) and (**b**) ammonium (NH_4_^+^) onto date palm waste derived biochar (BC), polyethylene (PE), polyamide (PA), BC amended with PE, and BC amended with PA.

The impacts of solution pH on the sorption of P and NH_4_^+^ using BC and MPs are shown in Figure 3. The sorption of P and NH_4_^+^ was significantly affected by the solution’s pH. Basic, neutral, and acidic pH levels were used to assess the efficiency of the sorbents for P and NH_4_^+^ removal, keeping a constant dose, temperature, and initial adsorbate concentration. Both adsorbates showed a similar trend in sorption with changing the solution pH. The highest sorption of both P and NH_4_^+^ was recorded at pH value 7, followed by pH values 5 and 9. The highest NH_4_^+^ sorption was exhibited by BC-PE (82.18 mg g^−1^), followed by BC-PA (71.27 mg g^−1^), and BC (64.22 mg g^−1^), while the lowest sorption was indicated by PA (7.85 mg g^−1^) and PE (11.72 mg g^−1^) at pH value 7. The higher PE sorption compared with PA could be due to negatively charged surfaces of PE, which could bind with cationic species of NH_4_^+^; therefore, PE alone and in combination with BC has sorbed a higher amount of NH_4_^+^. The results showed the highest sorption of P by BC-PA (66.60 mg g^−1^), followed by BC-PE (55.88 mg g^−1^) and BC (45.93 mg g^−1^) at pH value 7, while lowest sorption was exhibited by PA (8.89 mg g^−1^) and PE (5.99 mg g^−1^) at pH value 7. It has been established that H_2_PO_4_^−^ and HPO_4_^2−^ are the dominant species at pH 3–11 [42]. On the other hand, the pHpzc of BC was 10.01, indicating a net positive charge on the surface of BC [43]. Thus, the development of attractive forces between negatively charged P species and positively charged BCs has resulted in the highest removal of P. Moreover, the highest sorption of P by BC-PA could also be due to positively charged PA particles, which aided BC for P sorption, whereas PE particles were negatively charged and showed comparatively lower sorption than PA [34].

Due to a range of functional groups, higher surface area, and surface charges, BC has been established as a potential sorbent for P and NH_4_^+^ removal [44]. Generally, the removal of P and NH_4_**^+^** through BC-based sorbents is followed by electrostatic interactions, ligand exchange, complex formation, precipitation, physio sorption, and ion exchange [45]. However, to improve the performance of BC for both cationic and anionic species, researchers are modifying BC via various physical, chemical, biological, and mechanical means [34]. The combination of BC and MPs could enhance the performance of BC for P and NH_4_^+^ removal from aqueous solution by improving the surface functional groups, surface area, surface charges, and porosity. The current study’s findings indicated that the addition of MPs has significantly enhanced the sorption of P and NH_4_^+^ onto BC at all pH levels, with the optimum pH of 7. BC amended with PE has sorbed 21.64% higher P and 27.98% higher NH_4_^+^, compared with pristine BC at pH 7. Likewise, the addition of PA resulted in 44.98% higher P and 10.99% higher NH_4_^+^ sorption compared with BC alone. The improvement in the P and NH_4_^+^ sorption with BC-PE and BC-PA could be due to the combined benefits of MPs and BC.

#### 3.2.2. Kinetic Sorption Trials

**Figure 4:**
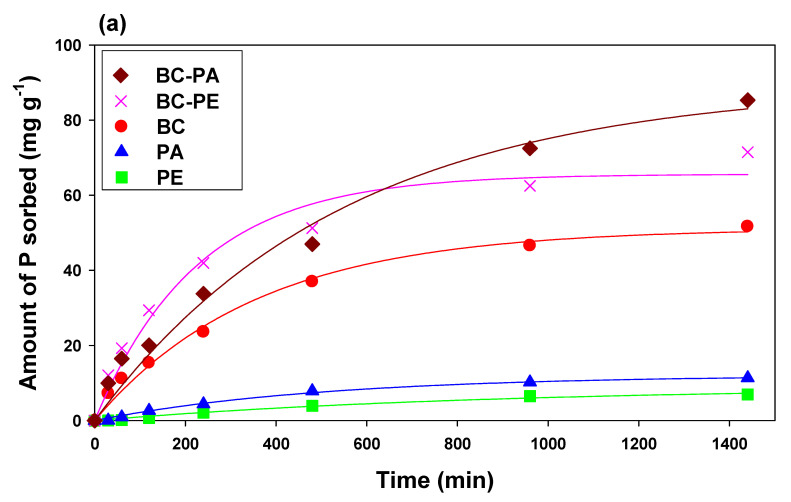

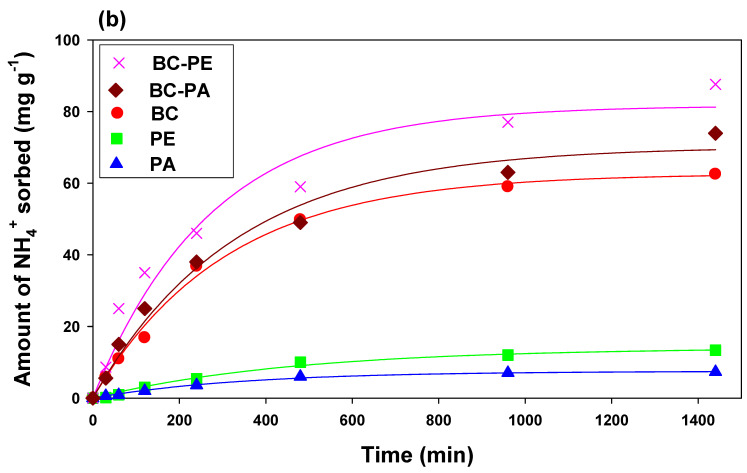
Sorption kinetics of (**a**) phosphorus (P) and (**b**) ammonium (NH_4_^+^) on date palm waste-derived biochar (BC), polyethylene (PE), polyamide (PA), PE amended BC, and PA amended BC.

The sorption kinetics of P and NH_4_^+^ onto BC and MP-amended BC were studied under constant temperature (23 ± 2 °C), pH, and initial sorbate concentration. The dynamics of P and NH_4_^+^ sorption are shown in Figure 4. Three stages were followed during the sorption of both the sorbates: rapid initial sorption, relatively slow, and finally equilibrium. The sorption was rapid at the beginning and declined with time as a consequence of more active site availability [46]. PE and PA exhibited the lowest efficacy and overall sorption of P and NH_4_^+^. After 200 min, the sorption of P and NH_4_^+^ was significantly reduced by PE and PA, which acquired equilibrium after 500 min. However, BC alone, as well as amended with MPs, showed relatively higher sorption of both P and NH_4_^+^. The rapid phase in BC and MPs amended BCs continued up to 400 min, resulting in higher P and NH_4_^+^ removal from the aqueous media. The highest P sorption was demonstrated by BC-PA, followed by BC-PE and BC (Figure 3a), while the highest NH_4_^+^ sorption was exhibited by BC-PE, followed by BC-PA and BC (Figure 3b).

The experimental sorption data were subjected to sorption kinetics modeling, as Table 2 presented derived parameters. The results showed that the sorption kinetics data were fitted to pseudo-first order, pseudo-second order, Elovich, intraparticle diffusion, and power function (*R*^2^ = 0.85–0.99). The pseudo-first-order and pseudo-second-order predicted sorption capacities (*qe*) for P were highest for BC-PA (4.33 mg g^−1^ and 78.02 mg g^−1^), BC-PE (3.94 mg g^−1^ and 60.61 mg g^−1^) and BC (3.92 mg g^−1^ and 58.13 mg g^−1^), respectively, whereas highest sorption capacities for NH_4_^+^ sorption was found in BC-PE (4.46 mg g^−1^ and 95.81 mg g^−1^) followed by BC-PA (4.33 mg g^−1^ and 84.05 mg g^−1^) and BC (4.08 mg g^−1^ and 74.11 mg g^−1^). A better fitting pseudo-first-order model of kinetic endorses P and NH_4_^+^ adsorption physically by tested adsorbents initially, while pseudo-second-order indicates chemical adsorption between adsorbate and adsorbent ions [47]. A similar trend of higher adsorption was found in Elovich model indicating highest sorption constant (*α*) for BC-PA (9.09) followed by BC-PE (8.07) and BC (7.34) to explain P adsorption by tested adsorbents while NH_4_^+^ sorption constant (*α*) was found highest in BC-PE (12.34) followed by BC-PA (10.43) and BC (9.51). The higher diffusion rate constant value (*k_id_*) of similar adsorbents explains the interlayer diffusion of adsorbate particles on the adsorbent, which indicates external mass transfer and engrossment of diffusion in the sorption mechanism [48].

#### 3.2.3. Equilibrium Batch Studies Isotherm

**Figure 5:**
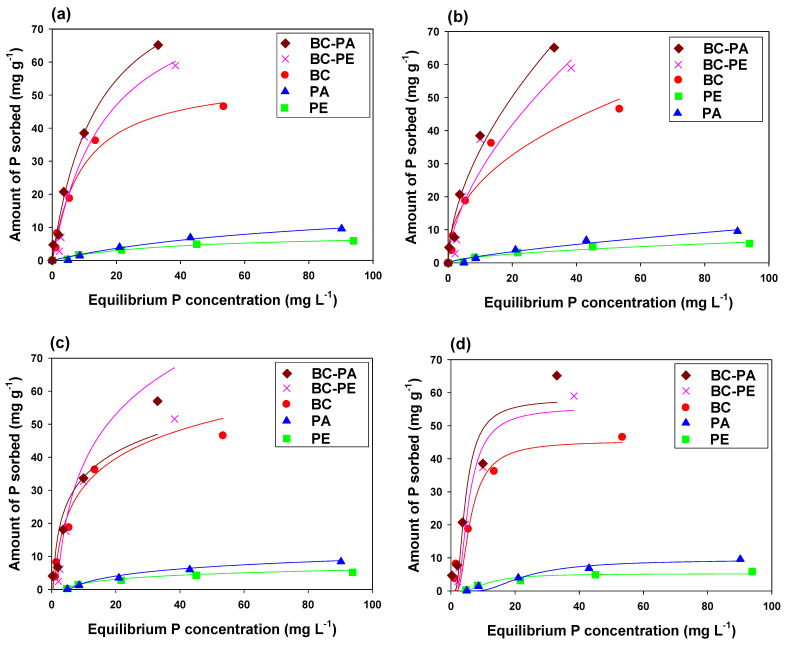
Phosphorus (P) sorption isotherm fittings on (**a**) Langmuir, (**b**) Freundlich, (**c**) Temkin, and (**d**) Dubinin–Radushkevich by date palm waste derived biochar (BC), polyethylene (PE), polyamide (PA), PE amended BC, and PA amended BC.

**Figure 6:**
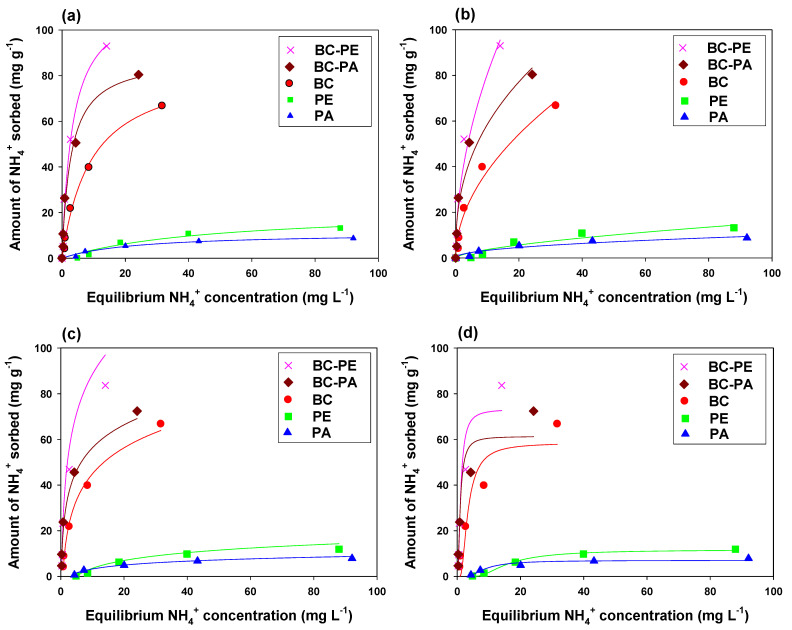
Ammonium (NH_4_^+^) sorption isotherm fittings on (**a**) Langmuir, (**b**) Freundlich, (**c**) Temkin, and (**d**) Dubinin–Radushkevich by date palm waste derived biochar (BC), polyethylene (PE), polyamide (PA), PE amended BC, and PA amended BC.

All the tested adsorbents were analyzed for P and NH_4_^+^ sorption, and sorption isotherm models were applied to interpret the sorption rate and mechanism (Figure 5 and Figure 6). Adsorption was conducted at variable adsorbent rates, and results concluded that by increasing adsorbate concentration, adsorbents showed more efficient adsorption. Since the adsorption process is highly dependent on initial adsorbate concentration, that is why initially adsorbents showed higher affinity for adsorbate due to more available active sites (H-type), which were gradually filled by adsorbate ions and adsorbent showed lower affinity for adsorbents later (L-type) explaining strong interaction between adsorbate and adsorbent. Composites of BC with PE and PA strongly influence P adsorption, and the highest P adsorption was found with BC-PA (93.23 mg g^−1^), BC-PE (86.81 mg g^−1^), and BC (54.08 mg g^−1^) (Table 3). The highest P sorption by BC-PA could be due to the positive surface charge on PA, which generated more positive ions on the BC surface and showed strong electrostatic interactions between P ions and BC, while comparatively low adsorption was found in BC, which could be due to net negative surface charge and repulsion between P ions and BC [34]. On the other hand, BC-PE (103.76 mg g^−1^) showed the highest sorption capacity for NH_4_^+^ ions, followed by BC (79.64 mg g^−1^) and BC-PA (85.83 mg g^−1^). The sorption of P and NH_4_^+^ was well fitted to applied Langmuir, Freundlich, Temkin, and Dubinin–Radushkevich (*R*^2^ > 90), which indicated a consistent physical layer adsorption initially, chemisorption and inner layer diffusion by adsorbents [46,49]. The Langmuir isotherm described a higher sorption capacity of P and NH**_4_^+^,** which indicated monolayer surface adsorption initially by mass transfer from the liquid phase to the solid adsorbent. Freundlich indicated the highest sorption constant (*K_F_*) for BC-PA (9.56 L g^−1^), which showed higher adsorption of P by PA followed by BC (9.12 L g^−1^) and BC-PE (7.56 L g^−1^), respectively. Nevertheless, BC-PE depicted the highest sorption constant for NH_4_^+^ (23.02), followed by BC-PA (20.94) and BC (11.61). Higher Freundlich constant values endorse higher sorption (chemisorption) on the heterogeneous surface of adsorbents. Additionally, the 1/*n* value for all tested adsorbents indicated Freundlich as a favorable isotherm model for all adsorbents [46,50]. Adsorbents presenting higher adsorption for P and NH_4_^+^ by previously applied isotherms also showed a similar trend of increasing adsorption by applying the Dubinin–Radushkevich isotherm. Likewise, BC-PA (58.04 mg g^−1^), BC-PE (55.60 mg g^−1^) and BC (45.39 mg g^−1^) showed highest affinity for P adsorption while highest NH_4_^+^ adsorption was found in BC-PE (73.24 mg g^−1^), BC-PA (61.29 mg g^−1^) and BC (56.48 mg g^−1^) by Dubinin–Radushkevich isotherm. Throughout the equilibrium and kinetics batch trials, consistently lower adsorption was found by MPs (without BC), which could be due to less available active sites and insufficient sorptive characteristics of PE and PA.

#### 3.2.4. Mechanism for P and NH_4_^+^ sorption

Experimental data indicated that P and NH_4_^+^ sorption were mediated by the initial pH of the aqueous solution and adsorbate initial concentration. In the beginning, sorption is favored by electrostatic interaction between opposite charge adsorbate ions and adsorbent surface. Also, electrostatic sorption endorsed physical adsorption on the adsorbent surface, which was also favored by the fitted Langmuir sorption isotherm. Eventually, the highest adsorption of P was found in PA-modified adsorbents (BC), which might be a result of more interactions due to electrostatic forces between negatively charged P (H_2_PO_4_^−^ and HPO_4_^2−^) ions and positive surface charge on BC-PA due to net positive charged PA surface. In comparison, a similar trend of higher initial adsorption was for NH_4_^+,^ with negatively BC-PE showing the highest sorption for positive NH_4_^+^ ions. Later on, owing to the porous surface structure of BC, these adsorbed ions were trapped in the surface and interlayer of adsorbents (BCs), which was also confirmed by intraparticle kinetics sorption. Previous research studies regarding P sorption on BC surfaces stated that mostly P ions were found adsorbed on the inner layer due to the porous nature of BC since both BC and P have a net negative surface charge, which is why fewer surface interactions were found between P ions and pristine BC [51]. Additionally, chemical precipitation between adsorbent and P ions was also involved in regulating chemisorption between adsorbate and adsorbent, which was also favored by the Freundlich sorption isotherm [52]. Additionally, the presence of MgO (Figure 1) also favored the chemical precipitation of P ions on adsorbents [53]. Also, owing to its porous surface and higher surface area, BC showed higher affinity and adsorption for P and NH_4_^+^ than MP (PE, PA) only treatments. Wang et al. [54], in a study, documented that the presence of surface functional groups enhanced NH_4_^+^ sorption, which indicated positive coordination between NH_4_^+^ ions and BC polar functional groups. Similarly, Bragmann et al. [55] and Spokas et al. [56] endorsed higher sorption of NH_4_^+^ due to the presence of surface functional groups against the surface porosity of the adsorbent. It also indicated that physio-sorption/surface sorption might not be the dominant mechanism for NH_4_^+^ adsorption. The abundance of polar functional groups on the BC surface endorse ion exchange between NH_4_^+^ and surface groups [57]. Our findings agree with Weldon et al. [58], who reported higher inter-layer diffusion of NH_4_^+^ ions on BC and stated intraparticle diffusion as a possible mechanism for NH_4_^+^ sorption.

## 4. Conclusions

This study was focused on MPs (PE, PA) assisted removal of P and NH_4_^+^ from an aqueous phase. The optimum pH for maximum adsorption of P and NH_4_^+^ was found to be 7, while the adsorption trend for P was found as BC-PA > BC-PE > BC >PA > PE, and for NH_4_^+^ it was found as BC-PE > BC-PA > BC > PE > PA. BC-PA showed higher sorption (93.23 mg g^−1^) for P against pristine BC (56.08 mg g^−1^), while PE (8.44 mg g^−1^) as well as PA (18.91 mg g^−1^) showed the lowest sorption of P. For NH_4_^+^, the lowest sorption was found in PA (10.15 mg g^−1^) and PE (19.30 mg g^−1^), while BC-PE (103.76 mg g^−1^) recorded the highest sorption, followed by BC-PA (85.83 mg g^−1^) and BC (79.64 mg g^−1^). Langmuir, Freundlich, and Temkin isotherms were the best-fitted adsorption isotherms and explained the chemisorption of adsorbate on tested adsorbents, while in kinetics studies, interparticle diffusion, pseudo-second-order, as well as power function models, explained the mechanisms of adsorption which suggested that chemisorption, electrostatic interaction, precipitation, diffusion, and ion exchange as possible mechanisms for P and NH_4_^+^ removal by date palm derived BC in the presence of PA and PE. The highest removal of both P and NH_4_^+^ was noticed in the presence of MP (PA and PE), suggesting a substantial contribution of MPs towards the sorption of P and NH_4_^+−^ from contaminated water. This study encourages the utilization of MP-loaded BC for the removal of P and NH_4_^+^ from contaminated water on a sustainable basis.

## Figures and Tables

**Table 1:** Chemical, proximate, surface area, and elemental composition analyses of the date palm waste biomass (BM) and its derived biochar (BC).

Property	Unit	BM	BC
Yield	%	–	31.04
pH	–	7.87	10.66
pH at point of zero charge (pH_pzc_)	–	7.25	10.01
Electrical conductivity	dS m^−1^	0.80	1.51
Moisture	%	6.83	2.52
Ash	%	8.40	27.12
Volatiles	%	82.11	24.28
Fixed carbon	%	2.64	46.07
Surface area	m^2^ g^−1^	1.62	260.05
Pore volume	cm^3^ g^−1^	0.009	0.145
Pore size	Å	226.38	22.43
C	%	45.51	63.72
O	%	48.40	34.92
H	%	5.56	0.58
N	%	0.53	0.78
O/C molar ratio	–	0.79	0.41
H/C molar ratio	–	1.45	0.10

**Table 2:** Parameters obtained from kinetic models for the sorption of phosphorus (P) and ammonium (NH_4_^+^) on date palm waste-derived biochar (BC), polyethylene (PE), polyamide (PA), PE amended BC, and PA amended BC.

Models	Parameters	P Sorption					NH_4_^+^ Sorption				
		BC	PE	PA	BC-PE	BC-PA	BC	PE	PA	BC-PE	BC-PA
First order	*k*_1_	1.7 × 10^−3^	1.3 × 10^−3^	1.7 × 10^−3^	1.6 × 10^−3^	1.8 × 10^−3^	1.9 × 10^−3^	1.9 × 10^−3^	1.6 × 10^−3^	1.8 × 10^−3^	1.9 × 10^−3^
	*R* ^2^	0.74	0.77	0.68	0.67	0.80	0.62	0.62	0.63	0.59	0.60
Second order	*k*_2_	−4.1 × 10^−5^	−4.9 × 10^−4^	−5.5 × 10^−4^	−2.6 × 10^−5^	−3.8 × 10^−5^	−4.9 × 10^−3^	−2.0 × 10^−3^	−1.9 × 10^−3^	−2.8 × 10^−3^	−2.7 × 10^−3^
	*R* ^2^	0.53	0.50	0.42	0.47	0.56	0.41	0.35	0.43	0.31	0.32
Pseudo-first order	*k*_1_′	2.6 × 10^−3^	1.2 × 10^−3^	1.7 × 10^−3^	2.5 × 10^−3^	2.6 × 10^−3^	2.9 × 10^−5^	5.3 × 10^−5^	5.4 × 10^−5^	2.7 × 10^−5^	4.5 × 10^−5^
	*q_e_*	3.92	1.58	2.11	3.94	4.33	4.08	2.52	1.75	4.46	4.33
	*R* ^2^	0.99	0.99	0.99	0.98	0.99	0.99	0.99	0.99	0.99	0.98
Pseudo-second order	*k*_2_′	5.8 × 10^−5^	5.5 × 10^−4^	1.0 × 10^−4^	8.3 × 10^−5^	4.0 × 10^−5^	4.0 × 10^−5^	7.9 × 10^−5^	2.0 × 10^−4^	4.0 × 10^−5^	4.4 × 10^−5^
	*q_e_*	58.13	6.08	13.88	60.61	78.02	74.11	19.73	10.18	95.81	84.05
	*h*	0.20	0.02	0.02	0.31	0.25	0.22	0.03	0.02	0.37	0.31
	*R* ^2^	0.99	0.95	0.86	0.99	0.95	0.99	0.85	0.90	0.99	0.99
Elovich	*a*	7.34	0.72	1.31	8.07	9.09	9.51	1.98	1.12	12.34	10.43
	*β*	−10.84	−1.14	−2.41	−9.64	−14.74	−15.07	−3.72	−1.91	−16.45	−15.94
	*R* ^2^	0.97	0.93	0.96	0.96	0.93	0.97	0.96	0.96	0.96	0.99
Intraparticle diffusion	*k_id_*	1.44	0.15	0.27	1.51	1.86	1.85	0.40	0.22	2.35	2.04
	*c*	0.56	−0.10	−0.56	4.20	−1.79	0.08	−0.91	−0.20	3.95	0.48
	*R* ^2^	0.98	0.99	0.98	0.96	0.99	0.92	0.96	0.93	0.96	0.97
Power function	*k_f_*	0.55	0.27	0.37	0.56	0.58	0.61	0.44	0.35	0.63	0.62
	*B*	0.07	−0.65	−0.84	0.25	0.06	−0.05	−0.90	−0.79	0.22	0.01
	*R* ^2^	0.99	0.99	0.97	0.97	0.99	0.95	0.95	0.95	0.91	0.93

**Table 3:** Parameters derived from non-linear isotherm models for the sorption of phosphorus (P) and ammonium (NH_4_^+^) on date palm waste derived biochar (BC), polyethylene (PE), polyamide (PA), PE amended BC, and PA amended BC.

Isotherms	Parameters	P Sorption					NH_4_^+^ Sorption				
		BC	PE	PA	BC-PE	BC-PA	BC	PE	PA	BC-PE	BC-PA
Langmuir	*Q_L_* (mg g^−1^)	56.08	8.44	18.91	86.81	93.23	79.64	19.30	10.15	103.76	85.83
	*K_L_* (L g^−1^)	0.12	0.03	0.01	0.06	0.07	0.12	0.02	0.04	0.30	0.36
	*R* ^2^	0.99	0.98	0.98	0.96	0.99	0.99	0.95	0.98	0.98	0.99
Freundlich	*K_F_* (L g^−1^)	9.12	0.53	0.40	7.15	9.56	11.61	0.79	1.02	23.02	20.94
	*1/n*	0.43	0.55	0.72	0.59	0.55	0.51	0.62	0.47	0.50	0.40
	*R* ^2^	0.93	0.95	0.96	0.92	0.98	0.97	0.90	0.93	0.95	0.95
Temkin	*b* (J mol^−1^)	215.67	1326.99	749.77	125.93	192.57	148.26	572.82	1061.61	116.10	161.91
	*A* (L g^−1^)	1.22	0.27	0.18	0.58	2.31	1.51	0.24	0.37	3.56	4.73
	*R* ^2^	0.98	0.99	0.99	0.99	0.85	0.99	0.98	0.98	0.99	0.99
Dubinin–Radushkevich	*Q_D_* (mg g^−1^)	45.39	5.22	9.52	55.60	58.04	58.48	11.68	7.03	73.24	61.29
	*E* (kJ g^−1^)	0.01	0.05	0.17	0.01	7.5 × 10^−3^	4.4 × 10^−3^	0.84	0.03	7.0 × 10^−4^	6.0 × 10^−4^
	*R* ^2^	0.93	0.91	0.96	0.97	0.91	0.88	0.99	0.93	0.91	0.91

## Data Availability

The data presented in this study are available from the corresponding author upon reasonable request.
